# Towards a metagenomics machine learning interpretable model for understanding the transition from adenoma to colorectal cancer

**DOI:** 10.1038/s41598-021-04182-y

**Published:** 2022-01-10

**Authors:** Carlos S. Casimiro-Soriguer, Carlos Loucera, María Peña-Chilet, Joaquin Dopazo

**Affiliations:** 1 Clinical Bioinformatics Area, Fundación Progreso y Salud (FPS), Hospital Virgen del Rocio, 41013 Seville, Spain; 2 Computational Systems Medicine, Institute of Biomedicine of Seville (IBIS), Hospital Virgen del Rocio, 41013 Seville, Spain; 3 Bioinformatics in Rare Diseases (BiER), Centro de Investigación Biomédica en Red de Enfermedades Raras (CIBERER), FPS, Hospital Virgen del Rocio, 41013 Seville, Spain; 4 ELIXIR-ES (INB), FPS, Hospital Virgen del Rocio, 41013 Seville, Spain

**Keywords:** Cancer, Computational biology and bioinformatics, Genetics, Microbiology, Systems biology, Diseases, Oncology

## Abstract

Gut microbiome is gaining interest because of its links with several diseases, including colorectal cancer (CRC), as well as the possibility of being used to obtain non-intrusive predictive disease biomarkers. Here we performed a meta-analysis of 1042 fecal metagenomic samples from seven publicly available studies. We used an interpretable machine learning approach based on functional profiles, instead of the conventional taxonomic profiles, to produce a highly accurate predictor of CRC with better precision than those of previous proposals. Moreover, this approach is also able to discriminate samples with adenoma, which makes this approach very promising for CRC prevention by detecting early stages in which intervention is easier and more effective. In addition, interpretable machine learning methods allow extracting features relevant for the classification, which reveals basic molecular mechanisms accounting for the changes undergone by the microbiome functional landscape in the transition from healthy gut to adenoma and CRC conditions. Functional profiles have demonstrated superior accuracy in predicting CRC and adenoma conditions than taxonomic profiles and additionally, in a context of explainable machine learning, provide useful hints on the molecular mechanisms operating in the microbiota behind these conditions.

## Introduction

In recent years the study of the microbiome has progressively gained interest, especially in the context of human health^1–4^. Microbial abundance profiles based on 16S rRNA genes have been used to study microbiomes, although whole genome sequencing (WGS) is becoming increasingly popular nowadays due to the decreasing sequencing costs^5,6^. Contrary to 16S rRNA data, WGS microbiome data provides the real gene composition in the bacterial pool of each sample, which allows identifying strain-specific genomic traits^7,8^. During the last years, microbiome WGS has been used to explore microbiome–host interactions within a disease context by means of metagenome-wide association studies, that allow studying gut microbiome alterations characteristic of different pathologic conditions^3,9–17^. In particular, recent evidence suggests that the human gut microbiome could be a relevant factor in human diseases^18,19^. In fact, the existence of carcinogenic mechanisms mediated by bacterial organisms has recently been proposed^20–22^. And, more specifically, it has been suggested that the gut microbiome could play a relevant role in the development of colorectal cancer (CRC)^15,16,23–25^. Due to this, the gut microbiome has been proposed as a potential diagnostic tool for CRC^16,17,26,27^. Nevertheless, its reproducibility and the predictive accuracy of the microbial gene signatures across different cohorts have been questioned^28,29^. The increasing availability of whole metagenome shotgun datasets of CRC cohorts^15–17,26,27^ facilitates large-scale multi-population exploratory studies of the CRC-associated microbiome at the resolution level of strain^30,31^. In two recent studies, a combined analysis of heterogeneous CRC cohorts was able to build accurate disease predictive models that open the door to the use of gut microbiota for future clinical prognostic tests^28,29^. The subsequent meta-analysis of the functional potential in the strains of the signature found gluconeogenesis and putrefaction and fermentation pathways associated with CRC, in coherence with the current knowledge on microbial metabolites implicated in carcinogenesis^32^.

It is important to note that the current approaches used to obtain biomarkers with predictive power use microbial strain or gene signatures as features to train a predictive model. Since genes or strains do not have a clear interpretability by themselves, the interpretation of the results of the classification produced by the model relies on the analysis of the potential functionalities encoded by these features. In other words, the predictive model is built using features that need to be interpreted a posteriori^33^. In fact, this is a relatively common problem with many current machine learning techniques, which have evolved in recent years to enable robust association of biological signals with measured phenotypes but, in many cases, such approaches are unable to identify causal relationships^34,35^. However, the interpretability of models, especially in a clinical context, is becoming an increasingly important issue^34–36^. The use of features with a direct functional interpretation has been suggested as crucial for the interpretability of the models^37^. In a recent study, gene profiles derived from WGS of samples of the MetaSub project^38^ were initially transformed into functional profiles, which account for bacterial metabolism and other cell functionalities, and have subsequently been used as features to build a city classification machine learning algorithm^39^. Since the features are informative by themselves, their relevance in the classification provides an immediate interpretability to the prediction model built.

Here, we propose an interpretable machine learning approach in which functional profiles of microbiota samples, with a direct interpretation, are first obtained from shotgun sequencing and subsequently used as features for predicting CRC in the patient donor of the sample. Moreover, in the prediction schema proposed, a feature relevance method allows extracting the most important functional features that account for the classification. Thus, any sample is described as a collection of functional modules contributed by the different bacterial species present in it, which account for the potential functional activities that the bacterial population in the sample, as a whole, can perform.

## Results

### Data-driven analysis of the interpretability

All the projects in Table 1 were preprocessed as described in methods and the corresponding taxonomic and functional profiles were obtained for all the samples. Supplementary Table S1 contains the list of taxonomic features, Supplementary Table S2 the list of KEGG functional features and Supplementary Table S3 the list of eggNOG functional features selected by the model. Also, Krona representations, allowing the exploration of these features at different hierarchical levels, are available for taxonomic (Supplementary Fig. S1) and KEGG (Supplementary Fig. S2) features, respectively.

**Table 1:** Datasets used in the study.

Project ID	Dataset name	References	Samples	Mean aligned reads
PRJNA389927	Hannigan	^40^	82	2.308.712
PRJEB12449	Vogtmann	^42^	104	3.897.639
PRJEB6070	Zeller	^16^	199	4.517.730
PRJEB7774	Feng	^15^	132	9.154.788
PRJEB10878	Yu	^17^	128	18.372.510
PRJNA447983	Thomas0	^29^	124	14.841.290
PRJEB27928	Thomas1	^29^	82	6.518.536

**Figure 1:**
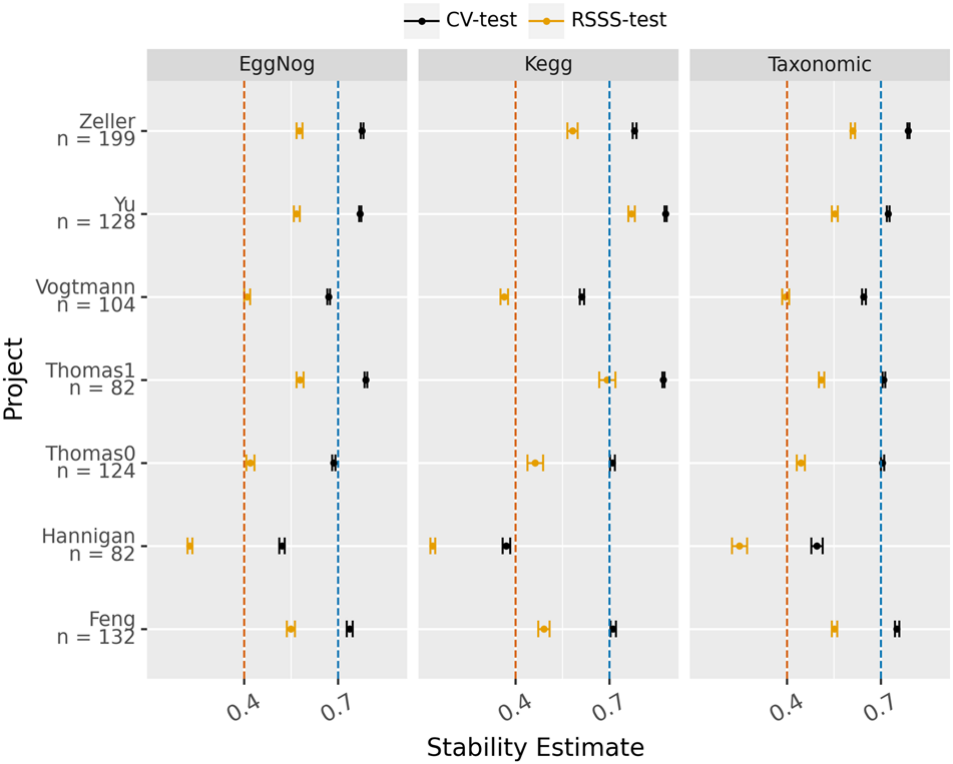
Stability estimate along with the confidence interval (alpha = 0.05) for the Random Stability Sub Sampling (RSSS-test) and 20-times tenfold cross validation (CV-test) splitting schemas, for each metagenomic profile (KEGG, eggNog and taxonomic) and project. The vertical bars indicate the theoretical thresholds on the effect size: below 0.4 represent bad agreement, between o.4 and 0.7 refers to a good enough agreement and scores above 0.7 represent a near perfect agreement.

**Figure 2:**
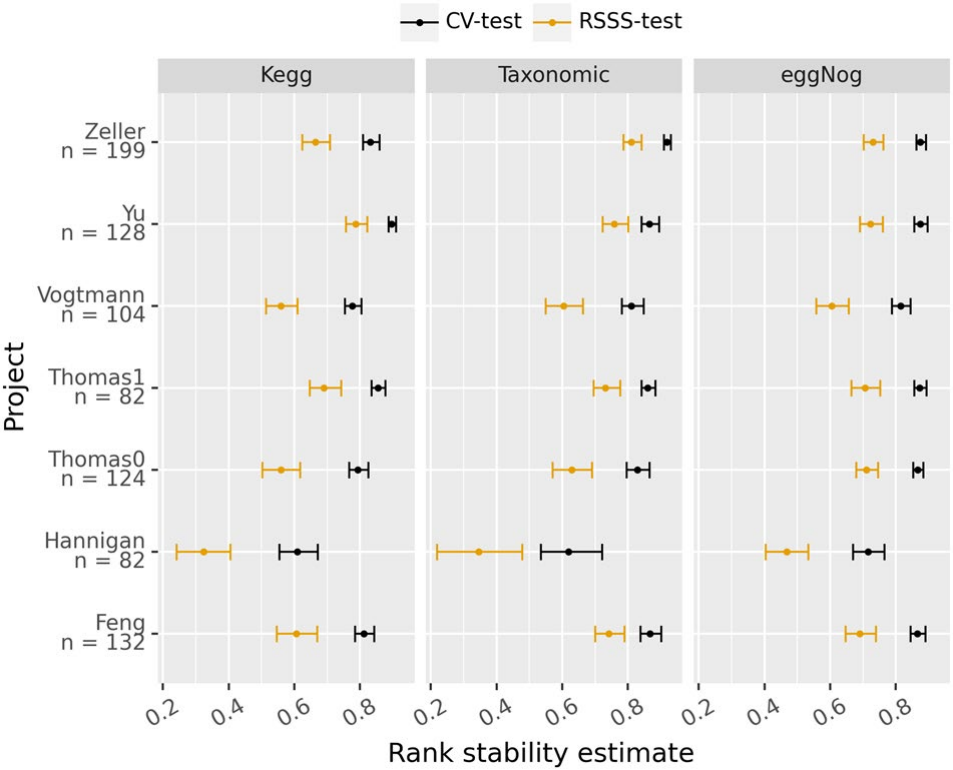
Rank stability estimate (the mean of all the hyperbolic-weighted tau pairwise rank comparisons) along with the 0.25 and 0.75 quantiles for the Random Stability Sub Sampling (RSSS-test) and 20-times tenfold cross validation (CV-test) splitting schemas for each metagenomic profile (KEGG, eggNog and taxonomic) and project.

**Figure 3:**
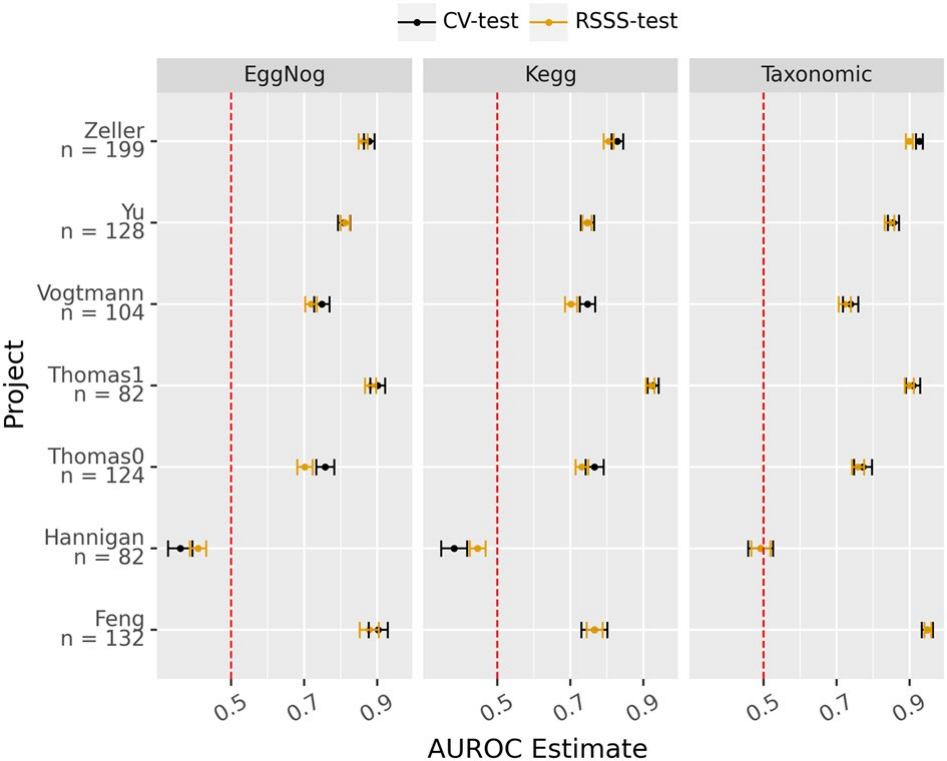
Mean of the area under the receiver operating characteristic curve (AUROC) along with the 0.25 and 0.75 quantiles for the Random Stability Sub Sampling (RSSS-test) and 20-times tenfold cross validation (CV-test) splitting schemas when discriminating between CRC and healthy samples for each metagenomic profile (KEGG, eggNog and taxonomic) and project.

A stability test was conducted for each project and profile used as described above and the results were congruent with previous observations: all the profiles are quite stable (stability score beyond 0.4 with small CI) except for the Hannigan project^40^, which happens to be the only project not sequenced at high depth (Fig. 1). In addition, the test for the cross-validation strategy was computed in order to detect the differences in the selection stability when training with more data. As can be seen in (Fig. 1), both analyses follow the same trend and, as expected, the larger the sample size, the greater the stability. The rank stability analysis (Fig. 2) follows the same pattern as the stability analysis which indicates that the features selected and their relevance are stable for each profile and experiment (Hannigan being again the exception) Note that the area under the receiving operating curve (AUROC) follows a similar trend, although not as pronounced, when the mean of the 20 repeated tenfold cross-validation strategy was compared with the average of the test splits over the stability splitting strategy (Fig. 3). Therefore, it can be concluded that under controlled experiments the greater the number of samples, the better the model performs.

### Performance analysis

A comparison of the method proposed here with those already published in the literature has been conducted using a performance validation schema previously proposed^29^, that consists of measuring the AUROC across the following data-splitting scenarios: (1) a 20-times repeated tenfold cross-validation for each project, (2) a cross-dataset prediction, which consists of training our model over one dataset to predict the rest, and (3) a leave-one-project-out (LOPO) design, where any given study is predicted with a model trained using the remaining projects.

However, the reference methodology conducts an out-of-training feature selection consisting of a two-step process that first preselects those features that are biologically more appropriate for gut-based microbiome analyses and secondly removes those features that are not statistically relevant (FDR correction of discrete correlations) using the whole dataset (the result of joining all studies). In order to have a fair comparison, we have performed two LOPO validation procedures: the first one does not perform any out-of-training feature selection (non-LOPO), thus leading to results more closely aligned to the original intent of checking the cross-dataset variations, while the second procedure uses an out-of-training feature selection (o-LOPO), making the model proposed here directly comparable to the reference methodology^29^.

**Figure 4:**
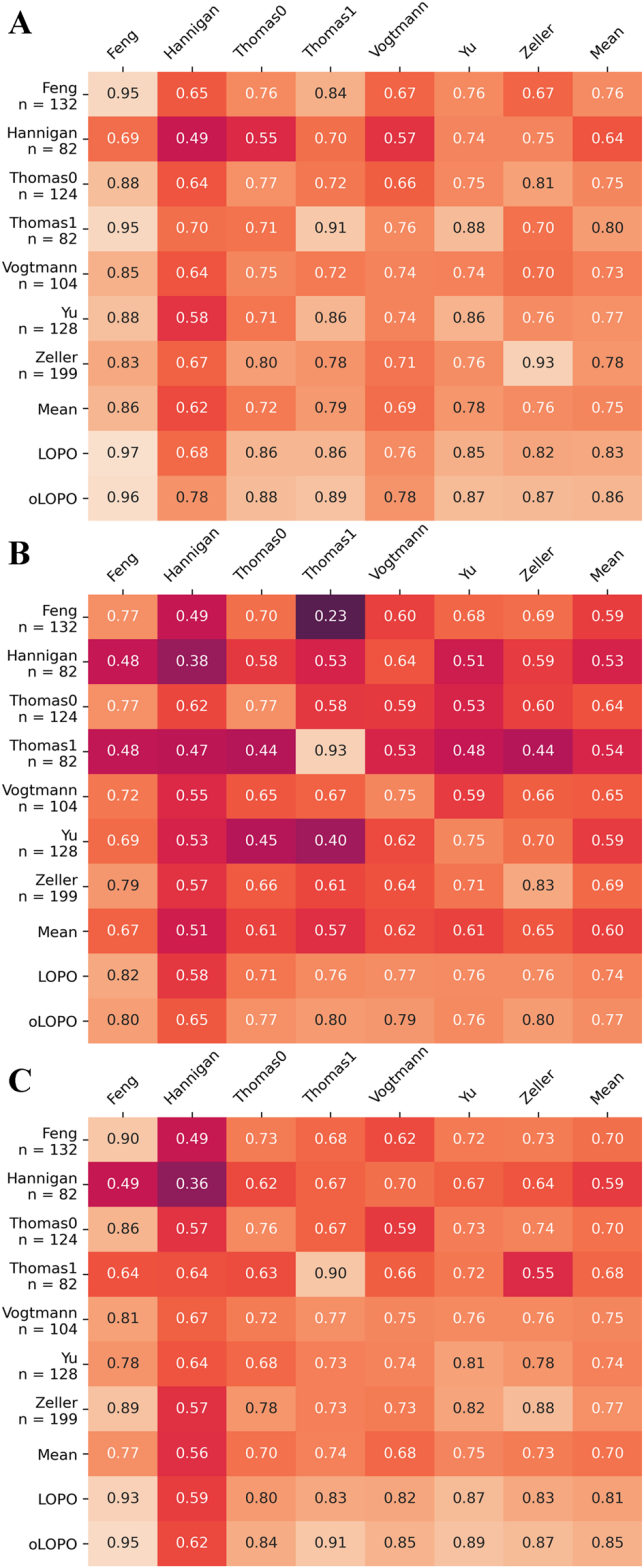
Cross-prediction matrix that measures the performance of the proposed model in terms of the area under the receiver operating characteristic curve (AUROC) for (**A**) taxonomic, (**B**) KEGG and (**C**) eggNog metagenomic profiles. The diagonal represents the intra-project performance by reporting the mean of the AUROC of 20-times tenfold cross validation, whereas the off-diagonal shows the cross-dataset performance, i.e. train with the model indicated in the rows and test over the project in the columns. Finally, the Leave one Project Out (LOPO) row reports the performance of predicting the dataset referred to in the columns while training with the other datasets, whereas the oLOPO row is the same experiment but using the functional signature learned during the LOPO procedure.

The results (Fig. 4) showcases: (1) the difficulties to generalize what the model learned in one dataset to others (non-LOPO off-diagonal scores), (2) a good intra-project performance (non-LOPO diagonal) except for the low-depth project, and (3) a good out-of-project performance that can be achieved by aggregating information from different projects. Overall, these results are congruent with previous observations^29^. Interestingly, we have found that the performance of the LOPO analysis in the model used here without pre-training is quite similar to the reference model^29^. However, when all the datasets are used for out-of-training feature selection the model used here behaves significantly better than the reference. These facts imply that not all the dataset-based signatures found by the model are shareable across the projects but rather a signature is learned by joining projects (increase in LOPO scores) as previously observed^29^. However, the model presented here makes a better use of increasing quantities of information: once noise has been filtered out through feature selection, the more depth in a profile the better the results.

### Signature computation and validation

For the interpretation analysis, a consensus signature has been built for each profile by combining the learned signature for each LOPO procedure as follows: (1) rescale the feature relevance for each LOPO run to [0, 1], (2) aggregate the score for each feature across all the runs and iii) divide it by (*p* − *nz(i)* + 1), where *p* is the number of projects and *nz(i)* is the number of projects where feature *i* is non-zero. Note that a feature that does not pass the FDR-based selection is assigned a score of 0.

**Figure 5:**
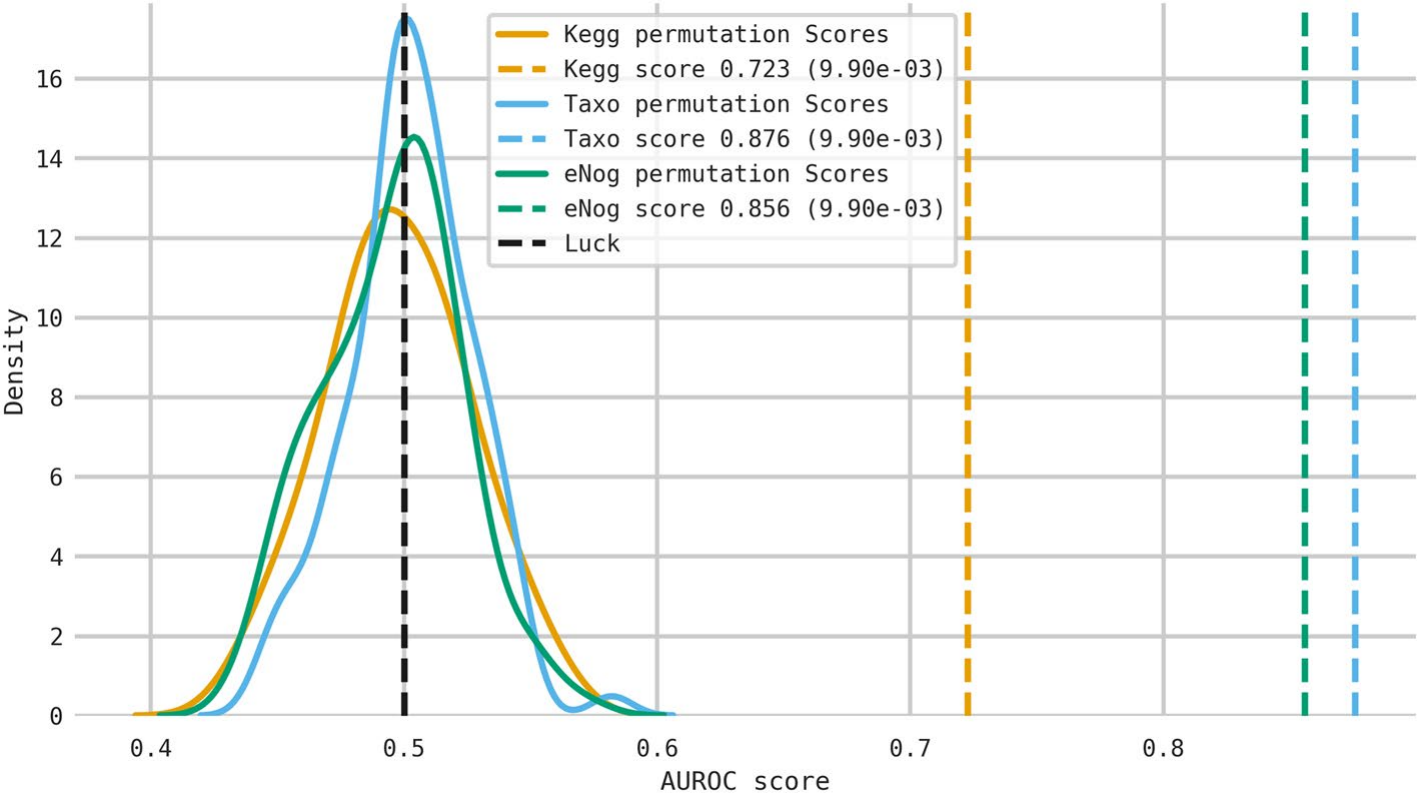
Significance of the cross-validated score through the use of the target permutation technique for each metagenomic profile (KEGG, eggNog and taxonomic). The p-value approximates the probability that the score for each profile would be the result of chance. The number of permutations is 100 for each profile using the consensus signature previously learnt and a 100-times tenfold cross-validation schema. Note that the worst outcome is 1 and the best is ~ 0.009. The vertical lines for each profile report the true score without permuting the outcome (being CRC or healthy) and the luck threshold (in black), whereas the continuous color lines show the permutation scores distribution (i.e. the null distribution) for each profile.

Finally, we have evaluated the significance of the o-LOPO scores for the consensus signature by means of the permutations tests technique technique^41^: for each profile we repeat the o-LOPO validation procedure for our pipeline 100 times while randomly permuting the outcomes. Then a p-value is computed by checking the percentage of runs where the trained model scored greater than the non-permuted score. As can be seen in Fig. 5 we can be confident that our model o-LOPO validation scores are significant (α = 0.05) for all the profiles. Note that the features where the pipeline is trained are fixed by keeping only those with a non-zero relevance score for the consensus signature.

### Adenoma analysis

In order to test how the proposed methodology can model unseen (but related) conditions a statistical test over the predicted probabilities of adenoma samples was proposed. The test procedure consists of the following steps: (1) a sample-wise concatenation of all datasets is carried out to construct three sets. All healthy and CRC (no comorbidities are considered here) samples are collected into the training set, which is further randomly divided into learning and validation sets with 0.7/0.3 the sample size. Finally, the test set is built with the samples not included in the previous sets (other diseases, adenomas and comorbidities including colorectal cancer). (2) The pipeline is first trained with the learning set and further used to compute the probabilities for all the samples of the validation and test sets. Then the distribution of the probabilities of the healthy samples is compared against the distribution of the adenoma samples that are not explicitly labeled as being small, using a Mann–Whitney rank test (healthy < adenoma). Other comparisons are also carried out, which include: the non-small adenoma distribution against the samples with a CRC condition (adenoma < tumor), the small adenoma versus non-small adenoma (small adenoma < adenoma) and healthy (small adenoma < healthy). (3) Steps (1) and (2) are repeated 100 times and the frequencies for the test being passed with a significance of < 0.05 (i.e. a hit ratio) are assessed. Note that the splitting criteria has been specifically chosen to be suitable for comparing such distributions, as the validation and test splits were built with data unseen for the model, thus forcing the independence between both sets and the split where the model has been fitted.

**Figure 6:**
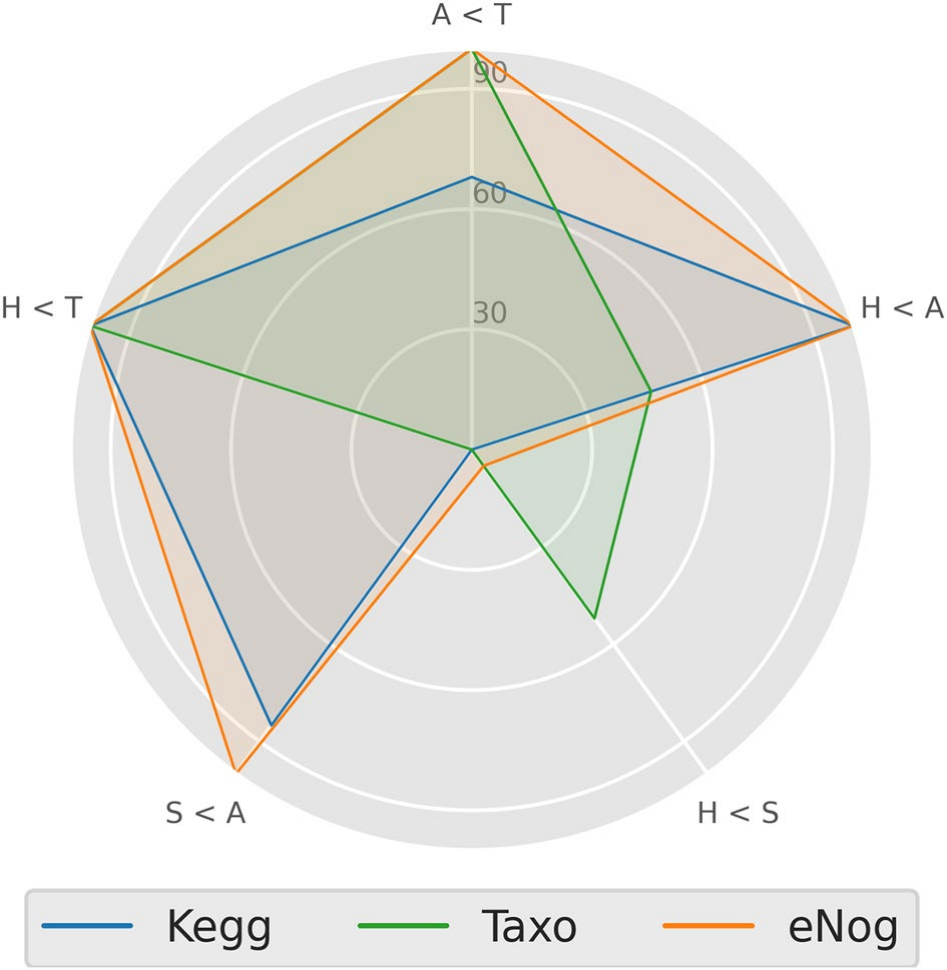
Radar plot with the performances of the different comparisons of the distribution of the probabilities between pairs of categories of samples using a Mann–Whitney rank test. Comparisons are clockwise: Adenoma < Tumor (A < T), healthy < Adenoma (H < A), healthy < small adenoma (H < S), Small adenoma < Adenoma (S < A) and healthy < tumor (H < T). Models were trained with Taxonomic (Taxo) and functional features (KEGG and eggNOG).

As depicted in Fig. 6 all the profiles perform well (100% hit rate) for the healthy < tumor test comparison. This is the expected behavior since we know from the performance analysis that all the profiles separate healthy from tumor. However, the eggNOG and KEGG functional profiles behave more like a risk score in terms of being CRC, due to the fact that the models based on those profiles assign a probability mass consistently higher to those samples more prone to having it (non-small adenoma) than healthy samples. Furthermore, the eggNOG profile achieves a 100% hit ratio for all the performed tests, except healthy < small adenoma. Thus, although the taxonomic profile and the eggNOG profile perform similarly from a healthy/CRC classification point of view, the former lacks the ability to see any difference between non-small adenoma and healthy samples, as evidenced by the hit ratio of the taxonomic profile in the healthy < adenoma test.

## Discussion

This study uses a comprehensive collection of the cohorts of CRC (listed in Table 1). Here, three different types of microbiome profiles (taxonomic and two functional ones based on KEGG and on eggNOG annotations) have been analyzed in an interpretable machine learning framework that has demonstrated to outperform other previous class predictors previously reported. As previously reported^16,29^, predictors render better predictions in the condition in which they were trained than in other conditions, independently of the type of profile used (see Fig. 4). The project PRJEB12449, which resulted with the worst performance, was frozen for more than 25 years before it was sequenced^42^. This most probably compromised the quality of the results^43^ and, actually, was described as technically flawed by previous studies^28,29^.

One of the most interesting properties of this approach is its immediate interpretability. Thus, the features chosen by the model that optimize the discrimination between the conditions compared account for the functionalities that operate differentially among both conditions.

At taxonomic level there are two species that clearly are relevant for the classification: *Parvimonas micra* and *Fusobacterium nucleatum*, which are represented by the four more relevant features (see Table 2). These two bacterial species have been related to CRC in numerous publications^44^ and are known CRC biomarkers^16,26,29^. In addition, *F. nucleatum* is known to promote chemoresistance in Colorectal Cancer cells by inhibiting apoptosis^45^ or by modulating autophagy^46^. Supplementary Table S1 lists the complete set of taxonomical features with the relevance assigned by the model. Actually, all the bacterial species listed in reviews as associated to CRC were selected by the model (some of them, like *Parvimonas*, *Fusobacterium*, *Porphyromonas, Gemella*, *Streptococcus* or *Clostridium* in Table 2 and the rest in preeminent positions in the relevance rank listed in Supplementary Table S1)^44^.

**Table 2:** The 20 most relevant taxons selected by the machine learning method used.

Relevance score	Name	Taxon ID
2.41252	*Parvimonas micra*	33,033
1.59494	*Fusobacterium nucleatum subsp. animalis 7_1*	457,405
1.56152	*Fusobacterium nucleatum subsp. animalis 4_8*	469,607
1.28725	*Fusobacterium nucleatum subsp. animalis*	76,859
0.96797	*Porphyromonas asaccharolytica DSM 20707*	879,243
0.81790	*Fusobacterium nucleatum subsp. nucleatum ATCC 23726*	525,283
0.70496	*Dialister pneumosintes*	39,950
0.62700	*Fusobacterium nucleatum subsp. polymorphum*	76,857
0.61251	*Gemella morbillorum*	29,391
0.57994	*Fusobacterium necrophorum subsp. funduliforme*	143,387
0.53820	*Clostridium sporogenes*	1509
0.53034	*Streptococcus anginosus C238*	862,971
0.50665	*Longibaculum sp. KGMB06250*	2,584,943
0.49567	*Anaerostipes hadrus*	649,756
0.47842	*Citrobacter freundii*	546
0.46420	*Fusobacterium nucleatum subsp. vincentii*	155,615
0.44508	*Streptococcus pseudoporcinus*	361,101
0.44365	*Blautia hansenii DSM 20583*	537,007
0.43355	*Fusobacterium nucleatum subsp. vincentii 3_1_36A2*	469,604
0.41181	*Fusobacterium nucleatum subsp. vincentii 3_1_27*	469,602

The analysis of functional profiles is even more interesting from the point of view of interpretability. Table 3 lists the 20 most relevant KEGG features selected by the model (see all the KEGG functional features in Supplementary Table S2). Interestingly, the most relevant feature is the *Methylaspartate mutase sigma subunit* (K01846), whose high activity is related to high probability of CRC according to the model. It has been described that cancer cells undergo modifications that include increased glutamine catabolism and over-expression of enzymes involved in glutaminolysis, including glutaminase^47^, which is liberated to the gut^48^ and promotes the proliferation of bacteria containing this bacterial module. Another known enzyme related to cancer present in Table 3 is *Heptose II phosphotransferase* (K02850). This enzyme, located in the *Lipopolysaccharide biosynthesis* (KO00540) pathway, is associated with CRC in high values. Actually, the presence of lipopolysaccharides produced in the surface of Gram- bacteria has been reported to induce an inflammatory response as well as to stimulate the proliferation of colon carcinoma^49,50^. Another relevant feature that discriminates healthy from CRC samples is *Manganese/zinc/iron transport system permease protein* (K11708). This transporter increases its number in excess iron conditions that are known to promote colorectal carcinogenesis^51^. *Methyltransferase* (K16168), related to polyketide synthesis, is the next most relevant feature. It has recently been described that a class of molecules, colibactins, are produced from the gene cluster called the *polyketide synthase island* that occurs in certain strains of *Escherichia coli* prevalent in the microbiota of CRC patients^52^.

**Table 3:** The 20 most relevant KEGG features selected by the machine learning method used here.

Relevance score	Name	KEGG ID
1.50544	glmS, mutS, mamA; methylaspartate mutase sigma subunit [EC:5.4.99.1]	K01846
1.34932	mal; methylaspartate ammonia-lyase [EC:4.3.1.2]	K04835
0.77486	rocR; arginine utilization regulatory protein	K06714
0.73183	pldB; lysophospholipase [EC:3.1.1.5]	K01048
0.73001	6GAL; galactan endo-1,6-beta-galactosidase [EC:3.2.1.164]	K18579
0.72130	pdaA; peptidoglycan-N-acetylmuramic acid deacetylase [EC:3.5.1.-]	K01567
0.71882	MARS, metG; methionyl-tRNA synthetase [EC:6.1.1.10]	K01874
0.70670	thiQ; thiamine transport system ATP-binding protein [EC:7.6.2.15]	K02062
0.70362	epr; minor extracellular protease Epr [EC:3.4.21.-]	K13277
0.66044	kamA; lysine 2,3-aminomutase [EC:5.4.3.2	K01843
0.62861	E2.1.1.77, pcm; protein-L-isoaspartate(D-aspartate) O-methyltransferase [EC:2.1.1.77]	K00573
0.62662	troC, mntC, znuB; manganese/zinc/iron transport system permease protein	K11708
0.62170	glpX; fructose-1,6-bisphosphatase II [EC:3.1.3.11]	K02446
0.61670	bpsB, srsB; methyltransferase	K16168
0.61284	spoIIP; stage II sporulation protein P	K06385
0.60627	waaY, rfaY; heptose II phosphotransferase [EC:2.7.1.-]	K02850
0.60596	FBA, fbaA; fructose-bisphosphate aldolase, class II [EC:4.1.2.13]	K01624
0.60594	rgpF; rhamnosyltransferase [EC:2.4.1.-]	K07272
0.59660	tex; protein Tex	K06959
0.58759	murA; UDP-N-acetylglucosamine 1-carboxyvinyltransferase [EC:2.5.1.7]	K00790

Metabolomic measurements in CRC also support the feature selection carried out by the model. A recent review on metabolic alterations in CRC provides a list of metabolites systematically altered in this cancer type^53^. Table 4 shows the metabolites most frequently reported as differentially deregulated in CRC. All of them are products of the KEGG orthologs selected by the model as most relevant features.

**Table 4:** Metabolites described as systematically deregulated in cancer and their relevance in the model using KEGG functional features.

Metabolite_name	HMDB_ID	KEGG_conpound_ID	Frequency	model_KEGG_KO_score
Glycine	HMDB0000123	C00037	24	4.823568715
L-Valine	HMDB0000883	C00183	23	0.6930878983
L-Alanine	HMDB0000161	C00041	22	3.22739351
L-Lactic acid	HMDB0000190	C00186	22	0.5279944356
L-Phenylalanine	HMDB0000159	C00079	20	2.117857375
L-Proline	HMDB0000162	C00148	20	1.434113835
L-Leucine	HMDB0000687	C00123	20	0.3006354234
L-Glutamic acid	HMDB0000148	C00025	17	13.32534903
Taurine	HMDB0000251	C00245	16	0.9103833031
Palmitic acid	HMDB0000220	C00249	15	0.3209053449
L-Methionine	HMDB0000696	C00073	15	4.503947812
Glycerol	HMDB0000131	C00116	14	1.125734858
L-Tyrosine	HMDB0000158	C00082	14	1.718910949
L-Threonine	HMDB0000167	C00188	14	1.354361221
L-Isoleucine	HMDB0000172	C00407	14	0.3873029906
L-Serine	HMDB0000187	C00065	14	2.042185229
L-Aspartic acid	HMDB0000191	C00049	14	3.752760624
D-Glucose	HMDB0000122	C00221	13	0.9079622305
L-Lysine	HMDB0000182	C00047	12	1.845182919
L-Arginine	HMDB0000517	C00062	12	1.818335229
L-Glutamine	HMDB0000641	C00064	12	5.074283424
Choline	HMDB0000097	C00114	11	0.3910553328
L-Asparagine	HMDB0000168	C00152	11	0.7764985602
myo-Inositol	HMDB0000211	C00137	11	0.3847705919
Succinic acid	HMDB0000254	C00042	11	1.819066672
L-Tryptophan	HMDB0000929	C00078	11	1.028295479
Acetic acid	HMDB0000042	C00033	10	4.986949589
Uridine	HMDB0000296	C00299	10	1.376520768

HMBD is the identifier of the metabolome database (https://hmdb.ca/) and the Frequency column denotes the number of studies in which the metabolite was found as deregulated according to a recent review^53^. The metabolite scores were calculated by adding the KEGG_KO’s scores, from the machine learning model, for each of the metabolites.

Another functional perspective is provided by the eggNOG features. Table 5 lists the 20 most relevant features (Supplementary Table S3 lists all the relevant eggNOG features). This type of features represents orthologous groups of proteins and constitute an interesting integration of function and taxonomy, given that protein families have a taxonomic-dependent distribution but, at the same time, play different roles in the bacterial biology^54^. Unfortunately, many bacterial proteins are still poorly annotated and about one third of the eggNOG features in the table are of unknown function. Interestingly, more than third are membrane proteins, which suggests that interaction of bacteria with the intestine cell could be playing a relevant role in CRC. Also, Methylaspartate mutase, E subunit, which correspond to two KEGG features with the best scores (Table 3).

**Table 5:** The 20 most relevant eggnog features selected by the model.

Score	Feature ID (eggNOG 4.5)	Taxonomic Level	Description
2.47062	08XIZ	bactNOG	Integral membrane protein TIGR02185
2.30583	06J4I	bactNOG	N/A
2.22012	0NI2F	firmNOG	Integral membrane protein TIGR02185
1.93081	00DN8	actNOG	One of the primary rRNA binding proteins, it binds directly to 16S rRNA where it nucleates assembly of the head domain of the 30S subunit. Is located at the subunit interface close to the decoding center, probably blocks exit of the E-site tRNA (By similarity)
1.92116	0NTFT	firmNOG	N/A
1.76985	0Y9D1	NOG	N/A
1.73496	0EX7J	cloNOG	N/A
1.47985	05DDE	bactNOG	Outer membrane autotransporter barrel domain-containing protein
1.46286	057E2	bacteNOG	DNA binding protein, excisionase family
1.4445	0587E	bacteNOG	Protein of unknown function (DUF1446)
1.37804	06F02	bactNOG	N/A
1.34625	05CMH	bactNOG	DEHYDRATASE
1.33923	08NTT	bactNOG	N/A
1.33323	079XJ	bactNOG	Major outer membrane protein
1.31216	08C1U	bactNOG	N/A
1.29914	08HM2	bactNOG	Cell wall binding repeat 2-containing protein
1.26147	059H0	bacteNOG	Methylaspartate mutase, E subunit
1.25394	05DCU	bactNOG	2-Hydroxyglutaryl-CoA dehydratase
1.24681	08BIB	bactNOG	Hypothetical bacterial integral membrane protein (Trep_Strep)
1.23689	07I7J	bactNOG	s-layer protein

Although an extensive description of the features selected by the model is beyond the scope of this paper, it is worth noting that the results obtained fully agree with the findings of functional analysis done in previous reports^28,29^.

Finally, a relevant aspect addressed in the study is the possibility of cancer interception by predicting CRC in early stages^55^. That would be the case of predicting adenomas. Actually, when the relative performance of the statistical test over the predicted probabilities of adenoma samples based either on functional or on taxonomic features is compared (Fig. 6) all the profiles distinguish tumors from normal samples with a 100% hit rate. However, functional profiles still show an excellent performance in distinguishing between CRC and adenoma samples and even adenoma from small adenoma samples, while taxonomic profiles fail to distinguish between these conditions. These observations suggest that the transition from normal condition to adenoma and CRC is not well defined in terms of strain abundances but there is a clear change at the level of functional activities of the bacteria in the sample that is better captured by functional profiles than by taxonomic profiles, which probably change at later stages, close to the CRC condition. This opens an interesting window of opportunity for clinical applications, as it has previously been suggested^28,29^, given that sequencing prices are plummeting to levels that obtaining taxonomic profiles result cost-effective in clinics. A trained predictor could systematically be used to detect in early phases individuals in risk of CCR. The results could be prospectively used to re-train the predictor.

Interpretability of the predictive models is becoming a major issue, especially in biomedicine^33,34,36^. The idea of using features with full biological meaning to gain interpretability in the machine learning methodology used has recently been proposed as a “white box” strategy^37^ and has successfully been used for the first time in the analysis of urban microbiota^39^ in the context of the METASub project^38^.

## Conclusions

The interpretable machine learning approach proposed here has demonstrated a more consistent performance in comparison to other approaches previously proposed when dealing with different CRC-based problems, while providing straightforward interpretations. Moreover, it demonstrated a better resolution not only with respect to the separation between healthy and CRC samples, but it is also able to discriminate samples with adenoma, being a promising tool for CRC prevention by detecting early stages in which intervention is easier and more effective. And finally, the model has a biological interpretation that provides important clues to better understand the mechanistic implications of the gut microbiota in CRC as well as in the previous stages of adenoma, which can have an interesting potential in preventive medicine and, specifically, in cancer interception^55^.

## Methods

### Data description

A total of 1042 fecal metagenomic whole genome sequencing (WGS) samples were analyzed. The samples were downloaded from the European Nucleotide Archive projects: PRJEB10878, PRJEB12449, PRJEB27928, PRJEB6070, PRJEB7774, PRJNA389927 and PRJNA447983. Sample metadata were obtained from the different supplementary tables of the corresponding publications^16,28,56^ and complemented in the possible using the R^57^ package *curatedMetagenomicData*^58^ available in Bioconductor^59^. Table 1 lists the experiments used in this study.

### Bacterial whole genome sequence data processing

Whole genome sequencing data was managed using the NGLess-Profiler^60^ package. Raw sequencing data preprocessing and quality control was carried out using a version of the *human-gut.ngl* pipeline. The *substrim* built-in function was used to discard reads that do not meet the basic quality filter of being longer than 45 bases and having all bases with a *Phred* score over 25. To prevent potential contaminations with human genome sequences the reads were mapped against the human genome hg19. All reads mapping the human genome were discarded. *SAMtools*^61^ and *BWA*^62^ were used to handle and map reads, respectively.

### Functional profiles

Strain functional profiles are generated by assessing the gene coverage for KEGG^63^ functional orthologs and eggNOG^54^ ortholog groups. Ortholog genes are the basic feature used here, and each sample is described as a vector of features, or feature profile. The representation of each feature of the profile in any sample is estimated from the number of reads mapping on the corresponding gene. These counts were obtained by mapping the reads that passed the filters mentioned above, using the integrated gene catalog of the human gut^64^. The NGLess built-in function *count* was used with the default values, applying the *scaled* normalization that consists of dividing the raw count by the size of the feature and then scaled up so that the total number of counts is similar to the total raw count.

### Taxonomic profiles

Strain taxonomic profiles were obtained using the *Centrifuge* application^65^. The *centrifuge-download* command was used to download the reference genomes of archea, bacteria, virus and vertebrate mammalian (human and mouse) taxons. The reads of each sample were mapped over the reference genomes. Taxons are here the features that describe each sample. The taxonomic profile consists of vectors composed by the relative representation of each genome (taxon) in the sample, which is obtained by normalizing the number of reads mapping on them by the respective genome lengths.

### Machine learning approach for tumor status prediction

For tumor status prediction a combination of classical machine learning techniques with a novel algorithm, the explainable boosting machine (EBM)^66–68^, were used for tumor status prediction with an aim towards interpretability. EBMs are state-of-the-art supervised learning ML whitebox models, also known as glassbox models, specifically designed for being highly explainable without losing predictive power.

The classification pipeline is sequentially constructed by concatenating the following methods: first the profile features are transformed using a logarithmic approach ($log\left( {1 + x} \right)$), then a feature selection based on the ANOVA F-test with FDR correction ($\alpha = 0.05$) is applied, followed by feature-wise discretization (2/20 bins for taxonomic/functional) and finally the EBM classifier is trained with the remaining features.

### Explainable boosting machine

Explainable boosting machines are a new type of Generalized Additive Models (GAMs)^69^, which are constructed by combining different ensemble-based techniques, such as bagging and boosting, with a feature learning algorithm that leads to highly interpretable models.

GAMs can be concisely written as:

$$g\left( {E\left( y \right)} \right) = \beta_{0} + \Sigma_{i} f_{i} \left( {x_{i} } \right)$$

where *g* refers to the link function (e.g. *logit* in classification problems), $y$ alludes to the outputs/labels and $f_{i}$ represents the (shape) function learned for each feature $x_{i}$. Traditionally the shape functions are either splines or polynomials^69^*,* which lead to interpretable models that lack predictive power^66^.

In the EBM algorithm each shape function is basically an ensemble of gradient boosted trees (GBT) constructed by iterating through all the features sequentially. For each feature a shallow tree is fitted, using only the selected feature, while the residuals are updated in a boosting-like fashion. Thus, each trained tree can only use the feature it was trained for, which allows the model to learn its contribution in a very precise way, while maintaining a *global* approximation by means of the residuals. These steps are repeated thousands of times by iterating through the data using such a small learning rate (GBT training) that the order of how the features are learned is not important. At the end, when all the iterations have been exhausted, the model builds a graph $f_{i}$ by aggregating all the trained trees for each feature, then the shape functions are combined together in order to assemble the final decision.

Despite the sophisticated of the learning procedure, the resulting model has all the intelligibility advantages of GAMs, since each shape function $f_{i}$ can be inspected in order to understand its contribution towards the final prediction. Furthermore, the repeated round-robin cycling over the features, along with the small learning rate, mitigates the effects of collinearity in feature-space (a common problem in biology), which leads to a fairer spread of the contributions ($\ell_{2}$- like)^67^.

### Explainability

The intelligibility of our model is driven by the combination of the different layers of the pipeline: the logarithmic preprocessing mitigates the skewness towards large values (frequent in biological multi project analyses), whereas the FDR-based selection drastically reduces the feature space by filtering out spurious relations with the outcome and, finally, the EBM learns a fair feature-attribution score, indeed a proper mathematical function (from now on, the *learning graph* or to simplify the *graph*), that fosters the biological interpretation of the problem.

As mentioned above, the EBM learns a *graph* for each feature and the learned representation can be very useful to complement decision-making in clinical scenarios due to the direct interpretability of the output^68^.

Moreover, the *graph* represents a visualization of the individualized feature contributions of each sample in the training dataset. Thus, the model constructs *global* (graphs) explanations on top of sample-wise explanations (*local*) without relying on external model-agnostic explainers, such as SHAP (SHapley Additive exPlanations)^70^ or local interpretable model-agnostic explanations (LIME)^71^. Note that a global relevance score can be computed by aggregating the absolute local attributions, which can be used to rank the feature importance for CRC prediction.

### Data-driven interpretability analysis

In order to check that the model delivers consistent results from an explainability point of view the stability of both, the feature selection and ranking methods of our pipeline were tested. Explanation-based performance tests are needed to account for the stochastic nature of the learning methodologies that drive both predictions and biological interpretations, the presence of technological and experimental noise, data sampling bias, etc.

On the one hand, the performance of the feature selection procedure is tested using the *Nogueira* stability measure and the associated statistical tests^72^, which consists of splitting any given dataset into 100 training and test subsets of half the total sample size, fitting the model on the training set while accounting for the features selected, and finally computing the stability measure using estimations of the variances of the selection of each feature. The final results are: (1) a stability score (SS) which ranges from 0 (random guessing) to 1 (perfect agreement), (2) a confidence interval for the score and iii) two theoretical thresholds on the effect size (below 0.4 represent bad agreement, up to 0.7 refers to a good enough agreement and scores higher than 0.7 represent a near perfect agreement).

On the other hand, the performance of the EBM relevance ranking method is tested using the hyperbolic-weighted tau (hwt) statistic^73^ which measures the correlation between a pair of rankings providing a good tradeoff between lessening the effects of the uninformative parts of a ranking and penalizing the deviations in the informative sections^74^. The test is a variation of Kendall’s tau, where the correlation between two rankings is corrected using an additive hyperbolic function that penalizes more the discrepancies on the top of the rank than those on the tail. The rank performance is estimated by building a distribution of all the possible hwt pairwise comparisons between the EBM rankings of each data partition (using the same splitting schema as in the stability analysis): a point estimate of 1 represents a perfect agreement, 0 is the score of two random uncorrelated rankings, whereas − 1 represents two opposite rankings.

### Software

The Machine Learning and statistical methods have been implemented in Python 3.7 on top of the *scikit-learn*^75^ (version 0.23.0), *Numpy*^76^ (version 1.18.4) and *SciPy*^77^ (version 1.4.1) libraries, whereas the EBMs have been trained and inspected using the *InterpretML*^78^ framework (version 0.1.22).

## Supplementary Information

Supplementary Information 1.

Supplementary Information 2.

Supplementary Information 3.

Supplementary Information 4.

## Data Availability

The datasets analyzed during the current study are available in the NCBI repository: PRJNA389927, https://www.ncbi.nlm.nih.gov/bioproject/389927. PRJEB12449, https://www.ncbi.nlm.nih.gov/bioproject/310722. PRJEB6070, https://www.ncbi.nlm.nih.gov/bioproject/266076. PRJEB7774, https://www.ncbi.nlm.nih.gov/bioproject/277324. PRJEB10878, https://www.ncbi.nlm.nih.gov/bioproject/297543. PRJNA447983, https://www.ncbi.nlm.nih.gov/bioproject/447983. PRJEB27928, https://www.ncbi.nlm.nih.gov/bioproject/486129. The code used in this work can be found at: https://github.com/babelomics/metagenomic-crc.
