# Supplementary material for: Towards a metagenomics machine learning interpretable model for understanding the transition from adenoma to colorectal cancer
Source: Sci Rep. 2022 Jan 10;12:450. doi: 10.1038/s41598-021-04182-y (PMC8748837; doi:10.1038/s41598-021-04182-y)
Supplement: Supplementary file 1 — Supplementary Information 1. [file 41598_2021_4182_MOESM1_ESM.pdf]

# Towards a metagenomics machine learning interpretable model for understanding the transition from adenoma to colorectal cancer

Carlos S. Casimiro-Soriguer, Carlos Loucera, María Peña-Chilet and Joaquin Dopazo

## Supplementary information

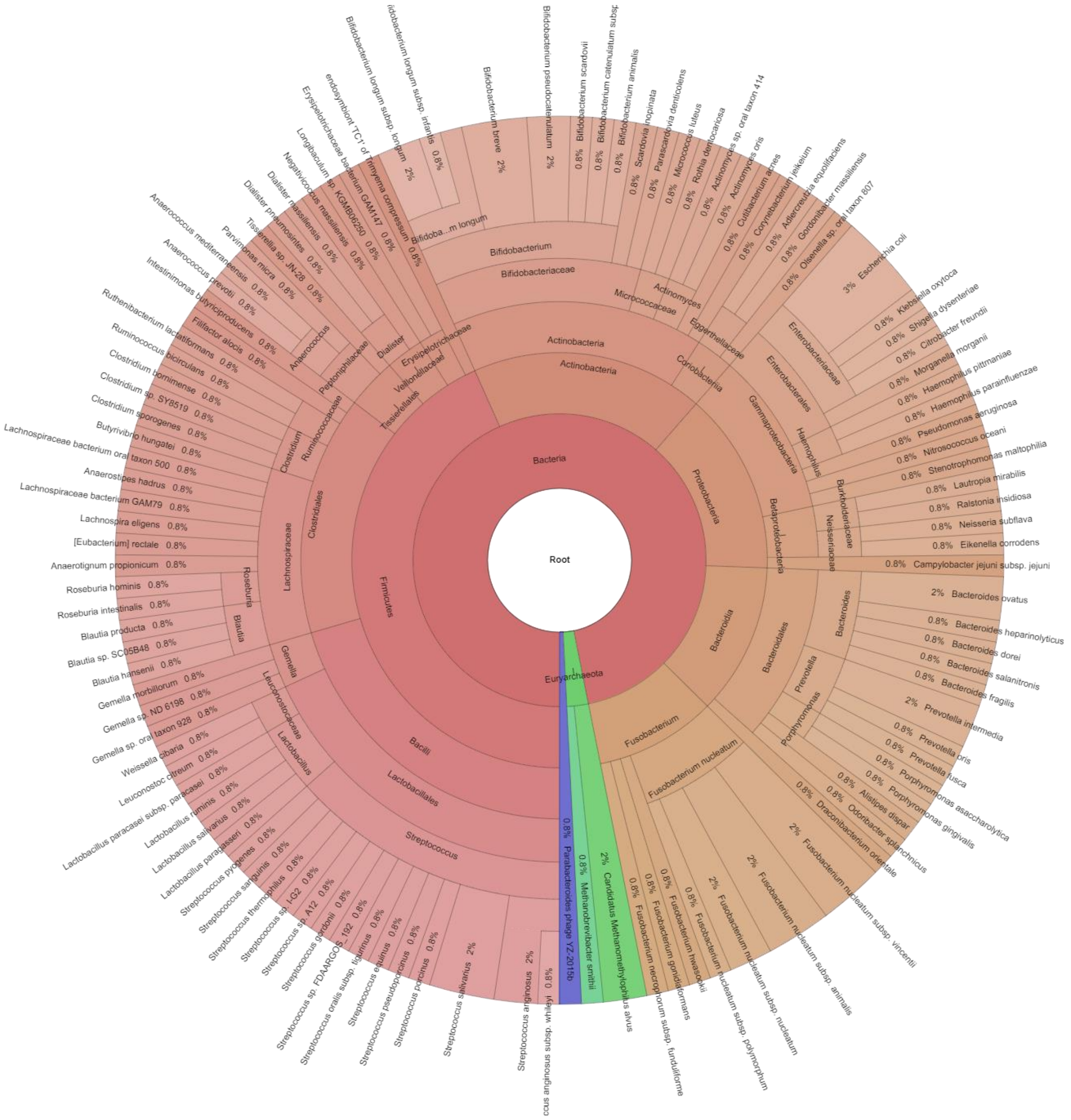


**Supplementary Fig. S1.** Krona representation of the most relevant taxonomic features selected by the model. An interactive version in Krona format is available at https://doi.org/10.5281/zenodo.4592917, **file** crc_collapse_norank.html.

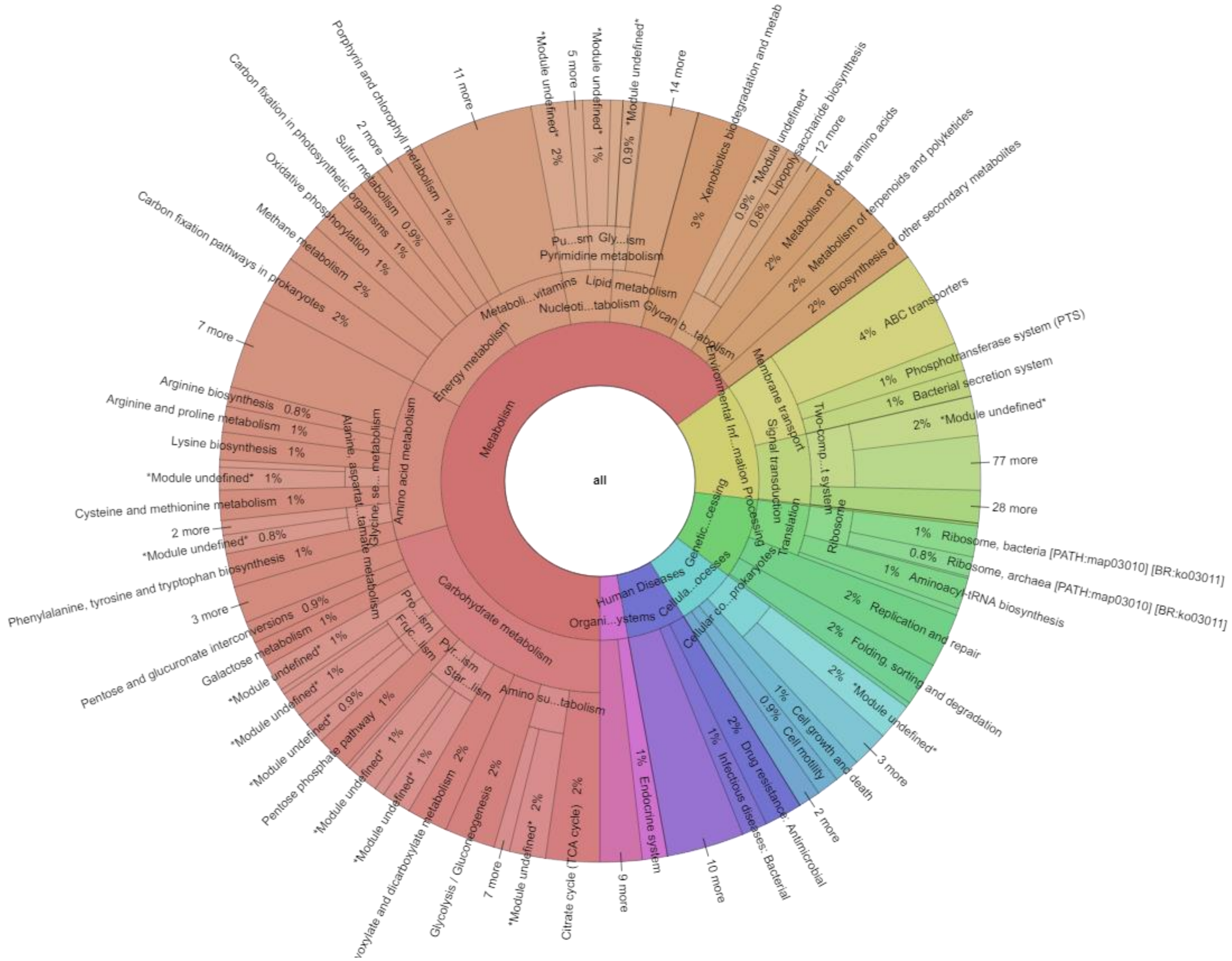


**Supplementary Fig. S2.** Krona representation of the most relevant functional KEGG features selected by the model. An interactive version in Krona format is available at https://doi.org/10.5281/zenodo.4592917, **file** crc_kegg_krona.html
